# Role, function, and expectations of research funding committees: Perspectives from committee members

**DOI:** 10.12688/f1000research.154665.1

**Published:** 2024-09-17

**Authors:** Amanda Blatch-Jones, Cherish Boxall, Katie Meadmore

**Affiliations:** 1 National Institute for Health and Care Research (NIHR) Coordinating Centre, University of Southampton, School of Healthcare Enterprise and Innovation, Southampton, England, S)16 7NS, UK; 2 Southampton Clinical Trials Unit, University of Southampton, Southampton, England, SO16 6YD, UK

**Keywords:** Funding committees, survey, virtual committees, grant applications, peer review, qualitative, expectations, research funding

## Abstract

Research funding committees play an integral role in the research funding process, consisting of a range of skills, knowledge, and expertise (e.g., professional, and public contributors). Although there is some evidence that has explored the efficiency and effectiveness of funding committees in terms of the funding process, there is a lack of published evidence about the purpose, role, and function of funding committees, from the perspective of committee members.

A subset of survey data from a cohort of six National Institute for Health and Care Research (NIHR) research programmes, exploring the purpose of a funding committee, and the expectations and role of a funding committee member between October 2020 to December 2021. All committee members were eligible to participate in the survey.

Using an inductive approach, 50 completed responses (22.5% response rate) were analysed, focusing on the role of a funding committee member and the functions of a funding committee. Participants highlighted seven key areas for the purpose of a funding committee: prioritising and recommending what research to fund (n=36) and assessment of quality (n=24) being the most common responses. Four areas were considered important to the expectations and role of funding committee members, with reviewing, critically appraising, and discussing applications (n=44); and being fair, objective, and unbiased (n=27) being the most common responses.

The findings offer a unique insight into committee members’ expectations about the role, purpose and function of a funding committee and their contribution to the funding recommendation process. There was high agreement that the purpose and role of committees and their members was to offer expert advice to make fair, impartial decisions on which research should be prioritised. Exploring the purpose, role, and function of funding committees has relevance and importance for funding organisations seeking to enhance and optimise the decision-making practice of funding committees.

## Introduction

Research funding committees (also referred to as grant panels or boards) are a core component of the peer review process. Peer review elicits expert, professional and public contributor opinions on whether to recommend or award a research proposal for funding. These committees play an important role for research funding organisations and aid in the funding recommendation and decision-making process. Abdoul *et al.* reported the role of the committee was to provide a clear summary of the proposal and the associated reviews, to act as an additional assessor and to establish a consensus decision. ^1^ However, there are variations between how funding organisations manage and conduct their funding committees; for example, some require decisions on which research proposals to award funding, whilst others provide recommendations on which proposals to fund, with the final decision laying elsewhere. ^2^ Several funding organisations provide terms of reference and guidance to support committee members’ understanding of their role, purpose and function as a member of a funding committee. For example, the National Institute for Health and Care Research (NIHR) provides information packs on their website for public contributors ( https://www.nihr.ac.uk/documents/public-committee-member-roles-information-pack-for-members-of-the-public/26580) and professional committee members ( https://www.nihr.ac.uk/researchers/have-your-say-on-our-research/become-a-professional-committee-member.htm).

Funding organisations may also differ in the number of peer review stages that they have, and the type of reviewer required (e.g., external review, funding committee review or community focused review). Some funding organisations use a two-stage application assessment process (stage 1 involves an outline application and stage 2 involves a full application), which involves external peer reviewing and funding committee review. It has been observed that the primary purpose of the committee at stage one is to assess the quality and value of the research question. By comparison, for stage two applications, the role of the committee is to decide which applications to recommend for funding. ^3^

The purpose and role of the funding committee may also influence the skills needed to make an effective committee member ^2^ and how the expectations and experiences of peer reviewers (including committee members) do not always align with the stated role and purpose of the committee. ^4^^,^^5^ Studies that have explored motivation and participation in funding committees demonstrate differences in these expectations. ^2^^,^^3^^,^^6^^–^^10^ For example, Gallo *et al.* identified that 87-92% of their participants felt that serving as a reviewer on a peer review panel had positively impacted their career, and this was influenced through improvements in writing grant applications and increased exposure to new scientific ideas compared to networking and collaboration opportunities. ^11^ These opportunities can unintentionally be seen to benefit or give greater advantage to those on the panel, potentially introducing the Matthew effect in research funding. ^12^

There has been considerable evidence addressing and analysing the research funding process, particularly in terms of efficiency and effectiveness of peer review by funding organisations. However, there is still a significant lack of evidence around the funding allocation procedures more generally and there are several reasons for this. ^13^ For example, Guthrie *et al.* and others have highlighted the sensitivity that surrounds funding organisations’ allocation and recommendations to fund research making this a particularly challenging context to undertake research. ^13^ Despite these challenges, understanding how the purpose, role, and function of funding committees is perceived by committee members themselves is integral to the research funding process, and can contribute to an important research evidence gap.

This paper addresses this important gap in the evidence and presents data from an online survey conducted as part of a larger study reported elsewhere that explored the role of virtual funding committee meetings for the allocation of NIHR research funding. ^3^ The aim of this paper is to focus on what funding committee members from several of the NIHR programmes consider the purpose of a funding committee to be, and what they consider the expectations of their role as funding committee member are. The insights drawn from the analysis provides a unique opportunity to explore the function and role of funding committees from a committee member perspective, that has not previously been explored in the research funding process literature.

## Methods

The survey data presented in this paper is a sub-sample from the main study exploring the NIHR virtual funding committee practices. ^3^ The purpose of the survey (as part of a netnographic study) was to gain further insight and understanding of virtual funding committees and explore the social interactions, expectations, and perceptions in a virtual setting of funding committee practice. The sub-sample addressed here, focuses on funding committee members expectations and role as a member of a committee and the purpose of a funding committee generally, from committee meetings conducted by the NIHR during October 2020 to December 2021 (for a detailed description of the full study, see Blatch-Jones *et al.,* 2023 ^3^).

### Study design

An online survey conducted as part of a netnographic study. This paper only reports on the questions from an online survey about the purpose, expectations and role of funding committees and their members and not specifically related to virtual or face-to-face committee meetings (see data analysis section for the two questions). All NIHR research programme funding committees that took place between October 2020 to December 2021 were eligible to participate in the study. A single NIHR funding committee was treated as a single online community.

### Data collection

All funding committee members from the research programmes who consented to participate in the netnographic study were sent a link to the survey within four weeks of the virtual funding committee taking place. The survey was open from October 2020 to January 2022. Due to the nature and sensitivity of funding committees and being able to identify respondents, no personal data were collected, unless they offered their contact details to be included in the interview part of the main study. All respondents were asked to complete an online consent form before they could access the survey. The survey was accessible for anyone with an internet connection and was held on a University of Southampton server, using Microsoft Forms. The survey included a total of 16 open and closed-ended questions, including Likert scale responses. We estimated from the pilot of the survey, it would take approximately 15-20 minutes to complete. All respondents were given three weeks to respond to the survey (with two reminders).

### Data analysis

For the analysis reported here, responses from two questions relating to the purpose, role, and function of NIHR funding committees and their members were analysed using an inductive (the data drove the coding of responses) qualitative content analysis approach. The survey data was downloaded from Microsoft Forms and moved to NVivo for analysis. These free text questions were:

1. As a member of a funding committee, what do you consider the purpose of a funding committee to be?
2. What do you consider the expectations and role of a funding committee member to be?

All responses were coded using Nvivo software (version 1.6.1). (A free alternative Computer-Assisted Qualitative Data Analysis Software (CAQDAS) to using Nvivo is Microsoft Excel or QualCoder that is open source ( https://qualcoder.wordpress.com/)) One researcher read through the responses for each question and created initial themes. The themes and associated quotes were then reviewed by a second member of the team, and theme names and associated quotes were then revised through discussion until consensus was met.

## Results

From a potential cohort of 222 invited committee members, 50 responses were received (response rate of 22.5%) from a total of six NIHR research programmes (further details are reported elsewhere ^3^). A high cloaking level was taken across all forms of data analysis, and where there was potential association to a funding committee or individual, verbatim quotes were amended from the online survey open-ended questions.

### Purpose of a funding committee

Several explanations describing the purpose of a funding committee were reported by the respondents (see Table 1 for a summary of responses). There was variation in how the respondents explained the purpose and role of a funding committee meeting; however, the majority of respondents thought that prioritisation; assessment of the quality of an application; and prioritising applications that answer questions of importance and relevance were key functions of a funding committee to ensure recommendations of research funding were for the best science that would maximise patient benefit. Although some respondents mentioned the importance of fair and transparent reviews, ‘value for money’ and feedback to applicants, prioritising the need and benefit to the NHS, staff, patients, and the public were seen as key factors during decision-making and form the core focus of discussion at these funding committee meetings.

**Table 1.** Qualitative content analysis of responses on the purpose of a funding committee.

Categorisation	No. of responses	Direct quotes
To prioritise and recommend which research to fund	36	“… ultimately decide whether the research should be funded.” “To recommend and prioritise research applications for funding and/or further information.”
Assessment of quality	24	“To assess the quality of funding applications and fund the best science.” “A fair and quality assessment (technical, ethical, value for money) of the proposal.”
To prioritise research questions of importance and relevance	21	“Allocation of funds to maximise benefit with respect to reducing uncertainty in healthcare/medical knowledge.” “… answering questions of clinical importance and that are relevant and important to patients.”
To provide expert advice and opinion through review and shared discussion with a diverse group of individuals	16	“To review research project proposals and share views of multidisciplinary team (clinicians, academics, statisticians, economist, patients).” “To provide expert opinions on funding applications to decide which applications should receive public money, determining need, feasibility and rigor of the research proposed. “
To provide fair and transparent review	7	“Make fair decisions to direct funding to the strongest research proposals which lie within the remit of the funding stream.” “To observe a fair and transparent process that enables public resources to be targeted at areas of genuine health (or social care) need.”
Assessment of value for money (e.g., cost effective)	7	“Assessing proposals for appropriateness, rigour, feasibility and likely value for money, and prioritising for funding.” “To ensure that applications meet a public need, are cost effective, methodologically sound and involve the public in all aspects of the project.”
Provide and offer feedback to applicants	5	“To provide feedback to applicants to improve the research plans to maximise its potential.” “For unsuccessful applicants, feedback to support their development.”

Based on the analysis of the responses from the online survey, several categories were discussed across the respondents’ responses. These are provided above along with the frequency of responses. For each category, quotes are provided to give an example of the responses.

### Expectation and role of a funding committee member

Exploring how respondents described their role as a member of a committee offered insight to what they expected from the role and whether this was comparable to how the purpose of a funding committee was reported (see Table 2 for a summary of responses). By contrast to the purpose of the funding committee as a whole, the majority of responses indicated that participants felt their role was to review and critically appraise applications and to discuss these views at the committee meeting. Several respondents mentioned how the role involves being able to provide an expert opinion and an objective and unbiased review (constructive criticism). Prioritising research applications according to clinical need and patient benefit was seen as important to the committee member role but this was not frequently mentioned compared to the purpose of the committee.

**Table 2.** Qualitative content analysis of responses on the expectation and role of a funding committee member.

Categorisation	No. of responses	Direct quotes
To review, critically appraise and discuss applications	44	“We are expected to present and/or comment on a specific set of proposals, and we are expected to have carefully reviewed these proposals because the rest of the committee will likely follow our suggestion. In addition, we are expected to revise all the other proposals, listen carefully to other presenters, contribute to the discussion and give a fair assessment to each of the proposals. “To review and critique research proposals; summarise and communicate strengths and weaknesses of the proposals to the committee.
To be fair, objective, and unbiased	27	“Provide an honest, unbiased and thorough review of funding applications highlighting strengths and weaknesses of each application.” “… to provide fair and objective comments around its strengths and weaknesses, to articulate clearly to other committee members the overall impressions of the application, to engage in meaningful dialogue/discussion about the applications to facilitate its fair assessment.”
To provide and share expertise in reviews and discussions	21	“To bring their strengths, skills and knowledge to bear on each application.” “Provide assessment of applications, based on the member's area of expertise.”
To prioritise and recommend funding	11	“… to assist the chair in recommending and prioritising research applications for funding/further information.” “To achieve consensus on funding where possible and to have sound and evidenced reasons for dissonance where necessary.”

Based on the analysis of the responses from the online survey, several categories were discussed across the respondents’ responses. These are provided above along with the frequency of responses. For each category, quotes are provided to give an example of the responses.

## Discussion

This paper provides a unique insight into the purpose, role, and function of funding committees from the perspective of funding committee members, from several programmes across the NIHR. Exploring these two aspects has provided new insights into what funding committees look for during the decision-making process but also how they see their role and function as part of the funding committee and in addition to funding committees’ terms of reference. Overall, from those that responded to the survey (22.5%) there was high agreement that the purpose and function of committees and their members was to offer expert advice to make fair, impartial decisions on which research should be prioritised for making funding decisions or make recommendations for funding.

Reassuringly, the roles of the committee reported were in line with general NIHR assessment criteria of the need for evidence, scientific rigour and value for money, and members perceived the main purpose of the committee meeting itself to be to prioritise and recommend which research to fund by assessing quality and ensuring the research is in areas of genuine need and clinical importance (based on NIHR guidance for committee members). Interestingly, value for money was equally valued the same as providing a fair and transparent review, which is often a feature in the feedback given to applicants. ^14^ Value for money is an important assessment criteria and is noted on the NIHR websites for committee members. In addition, the roles of the committee and its purpose also aligned to the NIHR terms of reference for joining a committee, including public contributors, demonstrating transparency from the NIHR in terms of the assessment criteria on how funding decisions are made by the funding committees.

Future work could explore the differences between roles, expectations, benefits, and motivations of a committee member to tease these important issues out. Better understanding of these may also allow funding organisations to create better strategies and incentives for recruiting to their committees and may also help to sustain transparency around funding committees and the decision-making recommendations of funded research for the research community as a whole.

### Strengths and limitations

The data reported in this paper come from two questions collected as part of a larger survey. Although the number of responses to the survey was acceptable, especially in the context of the wider study, a response rate of 22.5% is relatively low and the sample contained only those who were members of six NIHR funding committees. Although the sample only included one funding organisation, the findings incorporated several contexts by involving six NIHR programmes. ^13^ The data collection from 2020-2021 could also be seen as a limitation of the findings reported in this paper. Whilst the generalisability of our findings needs to be taken with caution, they provide a unique and reflective account on the purpose, role, and function of committee members that has not otherwise been explored previously. The findings add valuable contributions to the broader evidence around decision-making practices of funding organisations but more importantly addresses a gap in the evidence that goes beyond process measures and measuring effectiveness. ^13^

Variability in responses might have been due to different roles on the funding committee, terms of reference, committee inductions for new members and chairs, how long they have been a member and their professional position within academia, public community, clinical, or social care setting. Whilst the variability could also be an indicator of how funding committees are well briefed on the role and function as a member, it was not possible to explore this in more detail as no personal data were collected as part of the main study. A future study may wish to compare responses by exploring protective characteristics to better understand the different groups of people who join committees, and how these may in turn enhance the decision-making practices of funding committees and look across multiple funding organisations. To build on the existing evidence presented here from the perspective of the NIHR, a broader context from other funding organisations could generate more generalisable findings and comparative data.

## Conclusion

Funding committees play an important role in the decision-making process to fund research, yet there is no published evidence exploring their integral role, purpose, or function as committee members. The findings reported here contribute new evidence and insights about the value and importance of exploring funding committee members expectations and understanding of their role as members but also the function and purpose of a funding committee meeting. Although there was variation across respondents’ responses, there was clear consensus about what matters in order to make informed decision-making recommendations in relation to their role as a committee member but also how, as a collective group of experts, decisions are made that are fair and transparent, offering a considered and balanced discussion to make recommendations on what applications merit funding. Enhancing the current understanding in this space deserves more reflection, particularly from other funding organisations, to determine how and in what circumstances research applications are assessed, validated, and critically appraised to inform the decision-making process, whilst also appreciating the sensitivities around these funding allocation processes.

### Ethical considerations and consent

All respondents were asked to complete an online consent form before they could access the survey.

Consent from each participant of the survey was gained prior to completing the online survey. The study was approved by the University of Southampton, Faculty of Medicine Ethics Committee (ID 57541, 3 ^rd^ August 2020). This study was conducted according to the principles expressed in the Declaration of Helsinki.

## Data Availability

In line with our ethical approval, the survey data (alongside the interview and observational data) cannot be shared publicly to maintain confidentiality of our participants. Full details of the survey guide are provided in OSF: Exploring virtual funding committee practices in the allocation of National Institute for Health and Care Research funding: A netnographic study. https://doi.org/10.17605/OSF.IO/RZ6VT. ^15^ The project contains the following data:
• Appendix 4 Survey questions.docx Specified materials and guides are available under the terms of the CC0 1.0 Public domain dedication ( https://creativecommons.org/publicdomain/zero/1.0/). Additional requirements or requests are available from the corresponding author: A.J.Blatch-Jones@southampton.ac.uk) for researchers who meet the criteria for access to the data (e.g., researchers organisational affiliation, intended use of the data, and/or type of data). This main study is registered at RoR Registry and Hub (study number 805), accessible here: https://ror-hub.org/study/805/ **Reporting guidelines**: OSF repository. SRQR checklist for “Exploring virtual funding committee practices in the allocation of National Institute for Health and Care Research funding: A netnographic study.” ( https://osf.io/rz6vt/ ^15^). Data are available under the terms of the Crreative Commons Attribution International License (CC BY 4.0).
