# Supplementary material for: Role, function, and expectations of research funding committees: Perspectives from committee members
Source: F1000Res. 2025 Mar 6;13:1066. Originally published 2024 Sep 17. [Version 3] doi: 10.12688/f1000research.154665.3 (PMC11826072; doi:10.12688/f1000research.154665.3)
Supplement: Peer review history

# Reviewer report: Reviewer response for version 3

**DOI:** 10.5256/f1000research.178710.r369816

**Published:** 2025-03-11

**Authors:** Adrian Barnett

**Affiliations:** 1 Queensland University of Technology, Brisbane, Queensland, Australia

No further comments.

Is the work clearly and accurately presented and does it cite the current literature?

Yes

If applicable, is the statistical analysis and its interpretation appropriate?

Not applicable

Are all the source data underlying the results available to ensure full reproducibility?

No

Is the study design appropriate and is the work technically sound?

Yes

Are the conclusions drawn adequately supported by the results?

Partly

Are sufficient details of methods and analysis provided to allow replication by others?

Yes

Reviewer Expertise:

statistics, research funding

I confirm that I have read this submission and believe that I have an appropriate level of expertise to confirm that it is of an acceptable scientific standard.

# Reviewer report: Reviewer response for version 2

**DOI:** 10.5256/f1000research.176120.r365675

**Published:** 2025-02-13

**Authors:** Adrian Barnett

**Affiliations:** 1 Queensland University of Technology, Brisbane, Queensland, Australia

This paper has a clear question and is well reported. Funding panels are an understudied area relative to their importance, so evidence in this area is welcome. The study is relatively small in terms of sample size and the number of questions asked. Some results are fundamental, for example the commonly mentioned purposes in Table 1.

 A limitation that is not addressed is that neither of the two qualitative questions (page 4) prompted the reviewers to consider negative aspects of their experiences and/or systems changes that could be made. Hence the results may give an overly positive view of panels and the expectations of reviewers.

 Feedback was mentioned as a purpose, but is this also mentioned in the NIHR guidelines? And what feedback to applicants get?

 The small sample size may “introduce bias” (page 7), but it would be better to indicate the direction. The likely issue is that more interested and engaged panel members completed the survey, hence again the results may be slanted towards those who take their role more seriously.

 Citation number 3 can be updated from the preprint.

Is the work clearly and accurately presented and does it cite the current literature?

Yes

If applicable, is the statistical analysis and its interpretation appropriate?

Not applicable

Are all the source data underlying the results available to ensure full reproducibility?

No

Is the study design appropriate and is the work technically sound?

Yes

Are the conclusions drawn adequately supported by the results?

Partly

Are sufficient details of methods and analysis provided to allow replication by others?

Yes

Reviewer Expertise:

statistics, research funding

I confirm that I have read this submission and believe that I have an appropriate level of expertise to confirm that it is of an acceptable scientific standard, however I have significant reservations, as outlined above.

# Response

**Published:** 2025-02-25

**Authors:** Amanda Blatch-Jones

**Affiliations:** Faculty of Medicine, University of Southampton, Southampton, Hampshire, UK

**Comment 1: A limitation that is not addressed is that neither of the two qualitative questions (page 4) prompted the reviewers to consider negative aspects of their experiences and/or systems changes that could be made. Hence the results may give an overly positive view of panels and the expectations of reviewers.**

**Response: **Thank you for your comment. We have added additional detail of the potential bias in the data to the limitations section.

 Please note that this brief report only reports on two questions from a survey about the role, function and expectations of funding committee members. The full survey covered additional questions that did elicit both positive and negative responses. This  is reported in the full paper that can be found here: https://f1000research.com/articles/13-338

 Along with the supplementary material outlining the survey questions: https://osf.io/rz6vt/

**Comment 2: Feedback was mentioned as a purpose, but is this also mentioned in the NIHR guidelines? And what feedback to applicants get?**

**Response:** Applicants receive feedback from the panel committee as part of the funding allocation process. This is highlighted and explained in the NIHR guidelines. We have now added a paragraph to the discussion that discusses feedback.

**Comment 3:The small sample size may “introduce bias” (page 7), but it would be better to indicate the direction. The likely issue is that more interested and engaged panel members completed the survey, hence again the results may be slanted towards those who take their role more seriously.**

**Response: **Thank you. We have added some additional text to the revised version to reflect your comment.

 (those more interested and engaged could have been more likely to complete the survey)

# Reviewer report: Reviewer response for version 2

**DOI:** 10.5256/f1000research.176120.r356498

**Published:** 2025-01-22

**Authors:** Mario Coccia

**Affiliations:** 1 Arizona State University - Downtown Phoenix Campus, Phoenix, Arizona, USA; 2 CNR -- National Research Council of Italy, Collegio Carlo Alberto, Moncalieri, Italy

Role, function, and expectations of research funding committees: Perspectives from committee members

 The topics of this paper are interesting, though well known. The structure and content must be revised, and results have to be  better explained by authors before to be reconsidered.

 The **title** has to be shorter and should indicate the areas of these committees. 

**Abstract** has to clarify the goal and implications of research policy of results to improve decision process in funding.

**Introduction** has to better clarify the research questions of this study, indicating the gap presents in literature that this study endeavors to cover,  and provide more theoretical background. After that authors can focus on the topics of this study to provide a correct analysis for fruitful discussion (See suggested readings that must be all read and used in the text). 

**Methods **of this study can be clarified. The section of Materials and methods must be re-structured with the following three sections, clarifying the logic of the inquiry:

 •    Sample and data. Accurate description, also indicating the research fields and expertise. 

 •    Measures of variables

 •    Data analysis procedure. 

**Results**

 Authors have to avoid subheadings that create fragmentation and confusion. If necessary, can use bullet points (same comments for all sections). 

 I suggest also to represent some basic results with appropriate  graphical representation, such as bar graphs et. to be clear for readers.

**Discussion.** 

 First, authors have to synthesize the main results in a simple table to be clear for readers and then show what this study adds compared to other studies. 

 Although the Results section provides a detailed description, there needs to be a more critical synthesis and comparison of the findings with the literature. A discussion about possible biases in decision processes is important as well as considering the risk of failure in the research project funded, considering main errors to avoid. Authors  have to comment  on whether the results were expected for each set of findings; go into greater depth to explain unexpected findings. Moreover, either compare your results with the findings from other studies or use the studies to support these results. It is important to discuss how a gap in literature has been addressed. Expectations should be clarified on what it is based on and the logic of the decision process. Now the results are too vague and general. In general the expectations are associated with research fields and considering the overall ecosystem, also end users, if there are scarce resources or if there are emergencies (such as COVID) and committees can apply exaptation approaches, etc..., all these aspects have to be clarified. 

 Conclusion is slightly short. Conclusion has not to be a summary, but authors have to focus on manifold limitations (move here section presents in previous paragraph). Moreover, the Conclusion does not adequately discuss the theoretical and managerial implications of the study. Authors should claim for how the results can be applied more generally. They should clarify and systematize lessons learned in tables, proposing recommendations that can help improve a situation, or highlighting best practices.

 Make sure you create 3 subsections in the Conclusion: 1) Theoretical Implications, 2) Managerial or Policy Implications, and 3) Future Research.

 Overall, then, the paper is interesting, but theoretical framework is weak, and some results create confusion… structure of the paper has to be improved; study design, discussion and presentation of results have to be clarified using suggested comments.

 I strongly suggest improving the paper by using all comments (suggested papers are  included to be read and used all). 

 Suggested readings of relevant papers that have to be read and used in the text and references to improve theoretical framework, discussion and related implications for research policy.

 Schmaling, K.B., Evenson, G.R., Marble, B.K., Gallo, S.A. 2024 (Ref-1). Perceptions of grant peer reviewers: a mixed methods study. Research Evaluation, 33, rvae050    

 Gallo, S., Thompson, L., Schmaling, K., Glisson, S. 2018 (Ref-2). Risk evaluation in peer review of grant applications. Environment Systems and Decisions, 38(2), pp. 216–229    

 Coccia M. 2023 (Ref-3). New Perspectives in Innovation Failure Analysis: A taxonomy of general errors and strategic management for reducing risks. Technology in Society, vol. 75, n. 102384, https://doi.org/10.1016/j.techsoc.2023.102384

 Cristall, N., Drozdowska, B.A., Fladt, J., ... Goyal, M., Ganesh, A. 2024 (Ref-4). Priorities and expectations of researchers, funders, patients and the public regarding the funding of medical research: results from the PERSPECT qualitative study.BMJ Open, 14(11), e084655    

 Coccia M. 2024 (Ref-5). Theory of errors in innovation failure and strategic management of winning by failing. Technology Analysis & Strategic Management. https://doi.org/10.1080/09537325.2024.2383604

 Giannelos, K., Wiarda, M., Doorn, N. 2024 (Ref-6). Challenges to ethical public engagement in research funding: a perspective from practice.Open Research Europe, 4, 179    

 Ardito L., Coccia M., Messeni Petruzzelli A. 2021 (Ref-7). Technological exaptation and crisis management: Evidence from COVID-19 outbreaks. R&D Management, vol. 51, n. 4, pp. 381-392, https://doi.org/10.1111/radm.12455

 Schweiger, G., Barnett, A., van den Besselaar, P., ... Sandström, U., Conix, S. 2024 (Ref-8). The costs of competition in distributing scarce research funds    Proceedings of the National Academy of Sciences of the United States of America, 121(50), e2407644121 2024

Is the work clearly and accurately presented and does it cite the current literature?

Partly

If applicable, is the statistical analysis and its interpretation appropriate?

Partly

Are all the source data underlying the results available to ensure full reproducibility?

Partly

Is the study design appropriate and is the work technically sound?

Partly

Are the conclusions drawn adequately supported by the results?

Partly

Are sufficient details of methods and analysis provided to allow replication by others?

Partly

Reviewer Expertise:

science and research policy

I confirm that I have read this submission and believe that I have an appropriate level of expertise to state that I do not consider it to be of an acceptable scientific standard, for reasons outlined above.

## References

[1] : Perceptions of grant peer reviewers: a mixed methods study. *Research Evaluation* .2024;33: 10.1093/reseval/rvae050 10.1093/reseval/rvae050

[2] : Risk evaluation in peer review of grant applications. *Environment Systems and Decisions* .2018;38(2) : 10.1007/s10669-018-9677-6 216-229 10.1007/s10669-018-9677-6

[3] : New Perspectives in Innovation Failure Analysis: A taxonomy of general errors and strategic management for reducing risks. *Technology in Society* .2023;75: 10.1016/j.techsoc.2023.102384 10.1016/j.techsoc.2023.102384

[4] : Priorities and expectations of researchers, funders, patients and the public regarding the funding of medical research: results from the PERSPECT qualitative study. *BMJ Open* .2024;14(11) : 10.1136/bmjopen-2024-084655 e084655 10.1136/bmjopen-2024-084655 39609003 PMC11603686

[5] : Theory of errors in innovation failure and strategic management of winning by failing. *Technology Analysis & Strategic Management* .2024; 10.1080/09537325.2024.2383604 1-15 10.1080/09537325.2024.2383604

[6] : Challenges to ethical public engagement in research funding: a perspective from practice. *Open Res Eur* .2024;4: 10.12688/openreseurope.18126.2 179 10.12688/openreseurope.18126.2 39524111 PMC11549538

[7] : Technological exaptation and crisis management: Evidence from COVID‐19 outbreaks. *R&D Management* .2021;51(4) : 10.1111/radm.12455 381-392 10.1111/radm.12455

[8] : The costs of competition in distributing scarce research funds. *Proc Natl Acad Sci U S A* .2024;121(50) : 10.1073/pnas.2407644121 e2407644121 10.1073/pnas.2407644121 39621909 PMC11648638

# Response

**Published:** 2025-02-25

**Authors:** Amanda Blatch-Jones

**Affiliations:** Faculty of Medicine, University of Southampton, Southampton, Hampshire, UK

**The title has to be shorter and should indicate the areas of these committees.**

 The title is within the requirements for F1000Research brief reports.

 So as not to lengthen the title further, we have not added the funding committee research areas. The research areas have now been clearly articulated in the document (health and social care).

**Abstract has to clarify the goal and implications of research policy of results to improve decision process in funding.**

 The purpose of the brief report was to provide further clarification on two specific questions from the survey (that is reported in full elsewhere: https://f1000research.com/articles/13-338) as the role, function and expectations from the perspective of committee members is limited. The abstract provides insight for funders’ to ensure they optimise the decision-making practices for the allocation of funding.  

**Introduction has to better clarify the research questions of this study, indicating the gap presents in literature that this study endeavors to cover,  and provide more theoretical background.**

 Due to the limitations of brief reports (2500 words) we have briefly mentioned the relevant literature and have made it clearer that this article only focuses on the responses from the survey about how funding committee members answered two questions on the purpose and expectation and role of funding committee meetings. Paragraph 4 indicates the gaps in the evidence and why it is challenging to undertake research in the context of funding committee’s role and function.

 The full article provides greater detail, and the brief report is linked to that article (link provided above for convenience).

**Methods of this study can be clarified. Sample and data. Accurate description, also indicating the research fields and expertise.**

 Due to the sensitivity of the data and being able to identify members of committees and funding programmes, as reported in the article a high cloaking level was used to ensure anonymity of all committee members. As such we are unable to provide details of the individual committee members who took part in this survey. However, this does not impact the results due to being inclusive of all members and all members having equal contribution to the analysis and results.

**Results** **Authors have to avoid subheadings that create fragmentation and confusion. If necessary, can use bullet points (same comments for all sections).** **I suggest also to represent some basic results with appropriate  graphical representation, such as bar graphs et. to be clear for readers.**

 The data presented is qualitative due to the nature of the survey questions being open-ended and are not reporting any data that would be conducive to graphs. The results are provided under the two questions as stated in the introduction and methods section. The two tables offer insight to the qualitative content analysis, including examples of quotes from the two survey questions. This is a common way to present data from qualitative studies.

**Discussion. First, authors have to synthesize the main results in a simple table to be clear for readers and then show what this study adds compared to other studies. Although the Results section provides a detailed description, there needs to be a more critical synthesis and comparison of the findings with the literature. A discussion about possible biases in decision processes is important as well as considering the risk of failure in the research project funded, considering main errors to avoid. Now the results are too vague and general. In general the expectations are associated with research fields and considering the overall ecosystem, also end users, if there are scarce resources or if there are emergencies (such as COVID) and committees can apply exaptation approaches, etc..., all these aspects have to be clarified.**

 Following the brief report guidelines, we have provided a summary of what was presented in the brief report, and why it is relevant to existing literature. We also compared what was reported by committee members to the NIHR guidelines.

 Due to brief reports having a word limit of 2500, the guideline for a discussion includes “The results should be discussed in the context of existing literature. Strengths and weaknesses of the study should be presented and discussed. Future directions for work should be raised.” We have included the risk of potential bias under limitations and have included a statement about what direction this could be.

 This was a qualitative study that was exploring committee member perspectives of the purpose, role, and function of funding committees and committee members, and was analyzed in an inductive way. As such, had no prior expectations/framework of what committee members would report. In the discussion and within the word limitation, we have tried to discuss the findings in the context of prior literature where possible.

 Furthermore, due to the qualitative nature of the study, this means that the findings from this study cannot be generalised in the same way as other studies.  This has been mentioned in the limitations. However, we hope that overall learnings can be taken from the study and that it may prompt funders to reflect on their own funding committee practices.

 As described in the discussion, to date, there is very limited evidence that describes the purpose, role, and function of committees and their members from a committee member perspective. This study is therefore a step in addressing this gap.

**Conclusion is slightly short. Conclusion has not to be a summary, but authors have to focus on manifold limitations (move here section presents in previous paragraph). Moreover, the Conclusion does not adequately discuss the theoretical and managerial implications of the study. Authors should claim for how the results can be applied more generally. They should clarify and systematize lessons learned in tables, proposing recommendations that can help improve a situation, or highlighting best practices. Make sure you create 3 subsections in the Conclusion: 1) Theoretical Implications, 2) Managerial or Policy Implications, and 3) Future Research.**

 As this brief report is based data from two qualitative questions, it was not appropriate to extract generalizable recommendations.   Furthermore, as per brief report guidelines, a conclusion section is optional, but we have included this to offer some future considerations for the reader.  We hope that overall learnings can be taken from the study and that it may prompt funders to reflect on their own funding committee practices. As per brief report guidelines, a conclusion section is optional, but we have included this to provide context and future considerations for the reader. The full paper provides a more detailed conclusion based on your suggestions. However, in relation to this brief paper, we felt that it is not relevant to include these aspects. Thank you for suggesting them.

**Overall, then, the paper is interesting, but theoretical framework is weak, and some results create confusion… structure of the paper has to be improved; study design, discussion and presentation of results have to be clarified using suggested comments.**

 We have responded to your comments individually.

**Suggested readings of relevant papers that have to be read and used in the text and references to improve theoretical framework, discussion and related implications for research policy.**

 Thank you for your suggestions.

 Where appropriate we have added relevant references to explain how previous research has focused on specific areas, although there is limited evidence directly from committee members about what they perceive their role to be and the function of committee members. This particular focus is different to the assessment and process of peer review, review scores and decision-making through external peer review or panel/grant meetings.

 Peer review is not the feature of the article however some of the references have been added to further demonstrate the gap in the evidence. Thank you.

# Reviewer report: Reviewer response for version 2

**DOI:** 10.5256/f1000research.176120.r355325

**Published:** 2025-01-03

**Authors:** Santiago Vasco-Morales

**Affiliations:** 1 Universidad Central del Ecuador, Quito, Pichincha, Ecuador; 2 Neonatología, Hospital Gineco Obstétrico Isidro Ayora, Quito, Pichincha, Ecuador

This report evaluates an article on the research funding committees of the National Institute for Health and Care Research (NIHR). The article was restructured by including more details about the survey design and providing supplemental materials. The discussion of the results was deepened and the clarity in the presentation of the data improved. There are no more comments to make.

Is the work clearly and accurately presented and does it cite the current literature?

Partly

If applicable, is the statistical analysis and its interpretation appropriate?

Partly

Are all the source data underlying the results available to ensure full reproducibility?

Partly

Is the study design appropriate and is the work technically sound?

Partly

Are the conclusions drawn adequately supported by the results?

Partly

Are sufficient details of methods and analysis provided to allow replication by others?

Partly

Reviewer Expertise:

Pediatrics, neonatology, medical education

I confirm that I have read this submission and believe that I have an appropriate level of expertise to confirm that it is of an acceptable scientific standard.

# Reviewer report: Reviewer response for version 1

**DOI:** 10.5256/f1000research.169716.r341502

**Published:** 2024-11-25

**Authors:** Santiago Vasco-Morales

**Affiliations:** 1 Universidad Central del Ecuador, Quito, Pichincha, Ecuador; 2 Neonatología, Hospital Gineco Obstétrico Isidro Ayora, Quito, Pichincha, Ecuador

Summary of the Article

 This study explores the roles, expectations, and functions of research funding committees, focusing on the perspectives of committee members from six National Institute for Health and Care Research (NIHR) programs. Using data collected via an online survey, the authors identify key themes in the purpose of funding committees, such as prioritizing high-quality research, providing fair and unbiased reviews, and aligning decisions with public and clinical needs. The results contribute to the understanding of funding committee dynamics and highlight gaps in current practices, particularly regarding transparency and efficiency in decision-making.

 The study design employs qualitative content analysis to analyze responses, providing insights into the expectations and experiences of committee members. While the methodology is sound, several areas for improvement have been identified to enhance clarity, reproducibility, and scientific rigor.

 Detailed Review

 The following is an expanded evaluation of the article based on the key review questions:

 1. Is the work clearly and accurately presented, and does it cite the current literature?

 Answer: Partly

 Criticisms:

1. Integration with Literature: While the article cites relevant studies, it does not adequately connect its findings to the broader body of literature on peer review and funding committees.
2. Inconsistent Claims: The claim that there is no published evidence on the roles and expectations of funding committees is too broad. Relevant studies (e.g., Langfeldt, 2004; Lee, 2015) are mentioned in the peer review comments but not included in the discussion.

Recommendations:

 Incorporate additional references from the literature on peer review and funding committee practices to contextualize findings andsubstantiate claims.

 Expand the discussion section to critically compare findings with existing evidence, highlighting similarities and differences.

 2. Is the study design appropriate, and is the work technically sound?

 Answer: Partly

 Criticisms:

1. Survey Design: The survey questions are not fully described, limiting understanding of how data was collected.
2. Role Distinction: The study does not clearly distinguish between the roles of individual committee members and the collective purpose of the committee.
3. Low Response Rate: A 22.5% response rate may introduce bias and limit generalizability.

Recommendations:

 Provide a detailed breakdown of the survey design, including sample questions and their alignment with study objectives.

 Discuss the potential impact of the low response rate and how it may affect the representativeness of findings.

 Clearly separate analysis and discussion of individual versus collective roles.

 3. Are sufficient details of methods and analysis provided to allow replication by others?

 Answer: Partly

 Criticisms:

1. Lack of Transparency in Coding: Details on the coding process, inter-coder reliability, and how themes were finalized are not provided.
2. Limited Survey Details: The full set of survey questions is not included, and the categorization of responses lacks illustrative examples.

Recommendations:

 Describe the coding process in detail, including how consistency was ensured (e.g., inter-coder reliability metrics).

 Provide an appendix or supplementary materials with the full survey questionnaire.

 Include more examples of raw data (e.g., anonymized quotes) to demonstrate how responses were categorized.

 4. If applicable, is the statistical analysis and its interpretation appropriate?

 Answer: Not applicable

 Since the study focuses on qualitative content analysis, statistical analysis is not a core component. However, descriptive statistics, such as response frequencies, are used appropriately.

 5. Are all the source data underlying the results available to ensure full reproducibility?

 Answer: Partly

 Criticisms:

1. Supplementary Data: Additional resources, such as detailed coding frameworks or categorized data, are not provided.

Recommendations:

 Share anonymized or synthesized data to the extent possible, ensuring confidentiality.

 Provide coding frameworks and categorized themes as supplementary materials to support reproducibility.

 6. Are the conclusions drawn adequately supported by the results?

 Answer: Partly

 Criticisms:

1. Superficial Interpretation: While the conclusions are consistent with the results, they do not sufficiently explore broader implications or practical applications.
2. Generality of Claims: Conclusions could overgeneralize findings from a single funding organization to the broader context of research funding.

Recommendations:

 Deepen the discussion of how findings apply to funding committee practices and align with existing research.

 Acknowledge limitations in generalizability and provide recommendations for future studies across diverse funding contexts.

 Points That Must Be Addressed

 To make the article scientifically sound, the following points must be addressed:

1. Clarify Methods and Analysis:
  - Include detailed descriptions of the survey design and coding process.
  - Provide more illustrative examples of data categorization.
2. Improve Literature Integration:
  - Expand the discussion to connect findings with existing studies on peer review and funding committees.
3. Acknowledge Limitations:
  - Discuss the impact of the low response rate on findings.
  - Clearly state the limitations in generalizing results beyond the NIHR context.
4. Provide Additional Data:
  - Share supplementary materials (e.g., survey questions, coding frameworks) to enhance transparency and reproducibility.

Is the work clearly and accurately presented and does it cite the current literature?

Partly

If applicable, is the statistical analysis and its interpretation appropriate?

Partly

Are all the source data underlying the results available to ensure full reproducibility?

Partly

Is the study design appropriate and is the work technically sound?

Partly

Are the conclusions drawn adequately supported by the results?

Partly

Are sufficient details of methods and analysis provided to allow replication by others?

Partly

Reviewer Expertise:

porque Pediatrics, neonatology, medical education

I confirm that I have read this submission and believe that I have an appropriate level of expertise to confirm that it is of an acceptable scientific standard, however I have significant reservations, as outlined above.

## References

[1] : Fostering responsible research practices is a shared responsibility of multiple stakeholders. *J Clin Epidemiol* .2018;96: 10.1016/j.jclinepi.2017.12.016 143-146 10.1016/j.jclinepi.2017.12.016 29275217

[2] : A manifesto for reproducible science. *Nat Hum Behav* .2017;1(1) : 10.1038/s41562-016-0021 0021 10.1038/s41562-016-0021 33954258 PMC7610724

[3] : Meta-research: Why research on research matters. *PLoS Biol* .2018;16(3) : 10.1371/journal.pbio.2005468 e2005468 10.1371/journal.pbio.2005468 29534060 PMC5865753

# Response

**Published:** 2024-12-17

**Authors:** Amanda Blatch-Jones

**Affiliations:** Faculty of Medicine, University of Southampton, Southampton, Hampshire, UK

Thank you for your feedback. Please see the comments below: 

**Integration with Literature: While the article cites relevant studies, it does not adequately connect its findings to the broader body of literature on peer review and funding committees.**

 Thank you for your comment. We have revised the literature and added additional references.

**Inconsistent Claims: The claim that there is no published evidence on the roles and expectations of funding committees is too broad. Relevant studies (e.g., Langfeldt, 2004; Lee, 2015) are mentioned in the peer review comments but not included in the discussion.**

 We have revised the content of the article to address this point. Due to the limitations of brief reports (2500 words) we have briefly mentioned the relevant literature and have made it clearer that this article only focuses on the responses from the survey about how funding committee members answered two questions on the purpose and expectation and role of funding committee meetings. As per brief report guidelines, a conclusion section is optional, but we have included this to provide context and future considerations for the reader.

**Recommendations: Incorporate additional references from the literature on peer review and funding committee practices to contextualize findings and substantiate claims. Expand the discussion section to critically compare findings with existing evidence, highlighting similarities and differences.**

 Thank you. We have addressed your comment (and others) in relation to the wider literature and added in some additional text to the discussion. Due to the nature of brief papers, we did not go in depth as the survey complemented the wider netnographic study. As per brief report guidelines, a conclusion section is optional, but we have included this to provide context and future considerations for the reader.

**Survey Design: The survey questions are not fully described, limiting understanding of how data was collected.**

 Only two survey questions are related to this article (both reported under data analysis). Both questions are provided in the article. Links to the main study are provided. Data collection is included in the article, including timeframe and process of the complete survey.

**Role Distinction: The study does not clearly distinguish between the roles of individual committee members and the collective purpose of the committee.**

 In this brief paper, the two survey questions that are reported explore this distinction.  Q1 relates to the funding committee and Q2 to the individual, and Table 1 and 2 respectively show the findings for how the roles of the committee and the individuals are perceived by the funding committee members. We have made this distinction clearer in the discussion and added additional text comparing the role of committee members to the role of committees.

 In addition, due to the sensitivity of the data and being able to identify members of committees and funding programmes, as reported in the article a high cloaking level was used to ensure anonymity of all committee members. However, this does not impact the results due to being inclusive of all members and all members having equal contribution to the analysis and results.

**Low Response Rate: A 22.5% response rate may introduce bias and limit generalizability.**

 This is covered under strengths and limitations section.

**Recommendations: Provide a detailed breakdown of the survey design, including sample questions and their alignment with study objectives. Discuss the potential impact of the low response rate and how it may affect the representativeness of findings. Clearly separate analysis and discussion of individual versus collective roles.**

 This is reported in the brief paper but due to the 2500-word limit, providing the full details of the methods is not feasible, and it is reported elsewhere. We have reported the low response rate under the discussion section. It is not possible to report findings at an individual level, as reported above. However, we have made the distinction of the findings clearer in the discussion and added additional text comparing the role of committee members to the role of committees.

**Lack of Transparency in Coding: Details on the coding process, inter-coder reliability, and how themes were finalized are not provided.**

 A summary is provided in the brief paper. As stated, full details are reported elsewhere along with the wider themes of the main study. The purpose of the brief paper is to highlight the findings from two questions, as the current literature lacks evidence from real-world, first-hand understanding of what funding committee members perceive their role and function to be as a committee member and what the function and expectation is of a funding committee panel. As detailed, most of the literature is based on modelling, data, peer review, grant proposals, scoring etc. These studies do not address the two questions asked in the survey. By understanding the perceptions from committee members directly can help funding organisations enhance their guidance and consider where improvements are needed.

**Limited Survey Details: The full set of survey questions is not included, and the categorization of responses lacks illustrative examples.**

 These are reported in the main study and in the OSF repository as stated under the data availability statement. All materials are provided but F1000Research policy does not allow supplementary materials.

**Recommendations: Describe the coding process in detail, including how consistency was ensured (e.g., inter-coder reliability metrics). Provide an appendix or supplementary materials with the full survey questionnaire. Include more examples of raw data (e.g., anonymized quotes) to demonstrate how responses were categorized. Supplementary Data: Additional resources, such as detailed coding frameworks or categorized data, are not provided.**

 All relevant materials that comply with ethical procedures are provided in the OSF repository and linked to the main article as well.

**Recommendations: Share anonymized or synthesized data to the extent possible, ensuring confidentiality. Provide coding frameworks and categorized themes as supplementary materials to support reproducibility.**

 This is available on request as stated under data availability, to comply with ethical procedures, and to protect the identity of all participants. This is not a requirement for publication due to the sensitive nature of the data. However, we clearly state it is available on request.

**Superficial Interpretation: While the conclusions are consistent with the results, they do not sufficiently explore broader implications or practical applications.**

 We have revised sections of the brief paper to reflect your comments and feedback.

 As per brief report guidelines, a conclusion section is optional, but we have included this to provide context and future considerations for the reader.

**Generality of Claims: Conclusions could overgeneralize findings from a single funding organization to the broader context of research funding.**

 Thank you for your comment. This is noted under strengths and limitations. However, as with previous literature, most of the research is with one funder (several references listed demonstrate this point), and the context of each funding organisation will always be a limitation for all research studies. We have already stated this within the article.

**Recommendations: Deepen the discussion of how findings apply to funding committee practices and align with existing research. Acknowledge limitations in generalizability and provide recommendations for future studies across diverse funding contexts.**

 The netnographic study goes into depth on the aspects you mention. However, as noted this is a brief paper and is limited to 2500 words. Also, as per brief report guidelines, a conclusion section is optional, but we have included this to provide context and future considerations for the reader. The generalisability aspect is already noted in the article but we have extended a sentence to cover your comment.

**Points That Must Be Addressed: To make the article scientifically sound, the following points must be addressed:**

1. **Clarify Methods and Analysis:**
  - **Include detailed descriptions of the survey design and coding process.**
  - **Provide more illustrative examples of data categorization.**
2. **Improve Literature Integration:**
  - **Expand the discussion to connect findings with existing studies on peer review and funding committees.**
3. **Acknowledge Limitations:**
  - **Discuss the impact of the low response rate on findings.**
  - **Clearly state the limitations in generalizing results beyond the NIHR context.**
4. **Provide Additional Data:**
  - **Share supplementary materials (e.g., survey questions, coding frameworks) to enhance transparency and reproducibility.**

Please also see more detailed comments above that relate to these points and how we have addressed them. As described in the paper, please refer to the main study and the OSF repository, for a more comprehensive description of the whole survey design and analysis.

1. We have included additional references throughout the paper. However, due to the nature of brief papers, we did not go in depth on these.
2. Low response rate and generalisability are now covered under the strengths and limitations section.
3. All details and supplementary material are provided in the main study and the OSF repository. F1000Research policy does not allow supplementary materials.

 Thank you for your suggestions and detailed feedback.

# Reviewer report: Reviewer response for version 1

**DOI:** 10.5256/f1000research.169716.r328098

**Published:** 2024-10-23

**Authors:** Junwen Luo

**Affiliations:** 1 Economics, University College Cork Business School, Cork, County Cork, Ireland

This research is drawn from a small size survey which serves a larger research projects involving observations and interviews of the same pool of research funding reviewers. Overall, its research method, objective, and analysis are solid but can provide deeper thoughts and reflections in several ways.

 First, the quality and contribution of the paper will be improved if the authors could address how this paper’s conclusion is connected with the other aspects of the larger project as well as with the broad literature. For instance, are those reviewers’ internal expected roles and functions consistent with your observations of the committee meetings and especially the reviewer’s discussions and interactions on how to deal with conflicting views?

 By the way, some of the literature addressing such topics should be considered especially when you stated ‘there is no published evidence exploring their integral role, purpose, or function as committee members’, see below:

 Langfeldt, L. (2004). Ref 1

 Lee, C.J. (2015) Ref 2

 Langfeldt, L. (2001). Ref 3

 Luo, J., Feliciani, T., Reinhart, M.,.., & Shankar, K. (2021). Ref 4

 Van den Besselaar, P., and Leydesdorff, L. (2009) Ref 5

 Holbrook, J.B., and Hrotic, S. (2013) Ref 6

 Obrecht, M., Tibelius, K., & D’Aloisio, G. (2007).Ref 7

 Pier, E.L., et al. (2018) Ref 8

 Feliciani T, Luo J, Shankar, K (2022). Ref 9

 Feliciani, T., Morreau, M., Luo, J., Lucas, P., & Shankar, K. (2022)Ref 10

 Van den Besselaar, P., Sandström, U. & Schiffbaenker, H. (2018). Ref 11

 Second, for the analysis and results of content analysis, I would have expected more illustrations on your categorizations of the purposes and expectations which are your key findings, like how you derive those categorizations from the quotes and particular how DIFFERENT are those categorizations from each other? It’s also not very clear how you separated the expectations of the whole committee and of the individual reviewers in the study design, maybe through your survey questions settings?

 Third, an important aspect of the funding committee study, in my eyes, should be discussed which is the prorgamme objectives and target groups. This is found highly important for the committee discussion regarding how to align evaluation criteria into specific programme reviews and how to make fair and transparent grant reviews. A brief introduction of the programme that you studied should be provided in the Methods section. Discussion section could address more clearly how your findings can be connected to the existing literature (see above suggested ones) and make contributions to the broad area of research evaluation and peer review.

 Last but not least, I appreciate very much that the authors shared their research methods and guidelines including the survey questions that this paper made use of. For your reference, I am sharing another grant reviewer survey that you might find relevant and useful

https://doi.org/10.6084/m9.figshare.13651058.v1 (Shankar et al., 2021).

Is the work clearly and accurately presented and does it cite the current literature?

Partly

If applicable, is the statistical analysis and its interpretation appropriate?

Not applicable

Are all the source data underlying the results available to ensure full reproducibility?

Partly

Is the study design appropriate and is the work technically sound?

Yes

Are the conclusions drawn adequately supported by the results?

Partly

Are sufficient details of methods and analysis provided to allow replication by others?

Partly

Reviewer Expertise:

research evaluation, impact assessment, peer review

I confirm that I have read this submission and believe that I have an appropriate level of expertise to confirm that it is of an acceptable scientific standard, however I have significant reservations, as outlined above.

## References

[1] : Expert panels evaluating research: decision-making and sources of bias. *Research Evaluation* .2004;13(1) : 10.3152/147154404781776536 51-62 10.3152/147154404781776536

[2] : Commensuration Bias in Peer Review. *Philosophy of Science* .2015;82(5) : 10.1086/683652 1272-1283 10.1086/683652

[3] : The Decision-Making Constraints and Processes of Grant Peer Review, and Their Effects on the Review Outcome. *Social Studies of Science* .2001;31(6) : 10.1177/030631201031006002 820-841 10.1177/030631201031006002

[4] : Analyzing sentiments in peer review reports: Evidence from two science funding agencies. *Quantitative Science Studies* .2021;2(4) : 10.1162/qss_a_00156 1271-1295 10.1162/qss_a_00156

[5] : Past performance, peer review and project selection: a case study in the social and behavioral sciences. *Research Evaluation* .2009;18(4) : 10.3152/095820209X475360 273-288 10.3152/095820209X475360

[6] : Blue skies, impacts, and peer review. *https://riviste.unimi.it/index.php/roars/article/view/2914* .2013; 10.13130/2282-5398/2914 10.13130/2282-5398/2914

[7] : Examining the value added by committee discussion in the review of applications for research awards. *Research Evaluation* .2007;16(2) : 10.3152/095820207X223785 70-91 10.3152/095820207X223785

[8] : Low agreement among reviewers evaluating the same NIH grant applications. *Proceedings of the National Academy of Sciences* .2018;115(12) : 10.1073/pnas.1714379115 2952-2957 10.1073/pnas.1714379115 PMC586654729507248

[9] : Peer reviewer topic choice and its impact on interrater reliability: A mixed-method study. *Quantitative Science Studies* .2022;3(3) : 10.1162/qss_a_00207 832-856 10.1162/qss_a_00207

[10] : Designing grant-review panels for better funding decisions: Lessons from an empirically calibrated simulation model. *Research Policy* .2022;51(4) : 10.1016/j.respol.2021.104467 10.1016/j.respol.2021.104467

[11] : Studying grant decision-making: a linguistic analysis of review reports. *Scientometrics* .2018;117(1) : 10.1007/s11192-018-2848-x 313-329 10.1007/s11192-018-2848-x PMC613296430220747

# Response

**Published:** 2024-12-17

**Authors:** Amanda Blatch-Jones

**Affiliations:** Faculty of Medicine, University of Southampton, Southampton, Hampshire, UK

In response to your feedback please see the comments below: 

 First, the quality and contribution of the paper will be improved if the authors could address how this paper’s conclusion is connected with the other aspects of the larger project as well as with the broad literature. For instance, are those reviewers’ internal expected roles and functions consistent with your observations of the committee meetings and especially the reviewer’s discussions and interactions on how to deal with conflicting views?

**Response: **Thank you. We have extended the discussion to take account of your feedback.

 By the way, some of the literature addressing such topics should be considered especially when you stated ‘there is no published evidence exploring their integral role, purpose, or function as committee members’, see below:

 Langfeldt, L. (2004). Ref 1

 Lee, C.J. (2015) Ref 2

 Langfeldt, L. (2001). Ref 3

 Luo, J., Feliciani, T., Reinhart, M.,.., & Shankar, K. (2021). Ref 4

 Van den Besselaar, P., and Leydesdorff, L. (2009) Ref 5

 Holbrook, J.B., and Hrotic, S. (2013) Ref 6

 Obrecht, M., Tibelius, K., & D’Aloisio, G. (2007).Ref 7

 Pier, E.L., et al. (2018) Ref 8

 Feliciani T, Luo J, Shankar, K (2022). Ref 9

 Feliciani, T., Morreau, M., Luo, J., Lucas, P., & Shankar, K. (2022)Ref 10

 Van den Besselaar, P., Sandström, U. & Schiffbaenker, H. (2018). Ref 11

**Response: **Thank you for these additional references. Where appropriate we have added these references to explain how previous research has focused on specific areas, although there is limited evidence from committee members about what they perceive their role to be and the function of committee members. This particular focus is different to the assessment and process of peer review, review scores and decision-making through external peer review or panel/grant meetings.

 Second, for the analysis and results of content analysis, I would have expected more illustrations on your categorizations of the purposes and expectations which are your key findings, like how you derive those categorizations from the quotes and particular how DIFFERENT are those categorizations from each other? It’s also not very clear how you separated the expectations of the whole committee and of the individual reviewers in the study design, maybe through your survey questions settings?

**Response: **Thank you for your feedback. The survey data was at an individual level, and it is only this data presented in the brief report, as the full survey as part of the netnographic study is reported elsewhere.

 Under data analysis we explain the two questions used in this article, and the process we used.

 Third, an important aspect of the funding committee study, in my eyes, should be discussed which is the programme objectives and target groups. This is found highly important for the committee discussion regarding how to align evaluation criteria into specific programme reviews and how to make fair and transparent grant reviews. A brief introduction of the programme that you studied should be provided in the Methods section. Discussion section could address more clearly how your findings can be connected to the existing literature (see above suggested ones) and make contributions to the broad area of research evaluation and peer review.

**Response: **All NIHR programmes were invited to participate and six NIHR programmes participated in the netnographic study. To make this clearer all six NIHR programmes have been added in the results section for transparency purposes. Given the six NIHR programmes have different health focuses and aims, we have not included additional details or analysis on this. However, alignment to overall NIHR aims is discussed in paragraph 2 of the discussion.  We have revised the discussion section based on your feedback.

 As per brief report guidelines, a conclusion section is optional, but we have included this to provide context and future considerations for the reader.

 Last but not least, I appreciate very much that the authors shared their research methods and guidelines including the survey questions that this paper made use of. For your reference, I am sharing another grant reviewer survey that you might find relevant and useful

https://doi.org/10.6084/m9.figshare.13651058.v1 (Shankar et al., 2021).

**Response: **Thank you for sharing this survey on peer review.
